# National indication document and aortic valve replacement landscape in the Netherlands

**DOI:** 10.1007/s12471-023-01811-1

**Published:** 2023-10-16

**Authors:** Rob Eerdekens, Gijs van Steenbergen, Mohamed El Farissi, Jesse Demandt, Marcel van ’t Veer, Edgar Daeter, Leo Timmers, Arend de Weger, Niki Medendorp, Pim Tonino, the Transcatheter Heart Valve Intervention Registry Committee, B. van den Branden, M.M. Vis, W. A. L. Tonino, N. M. D. A. van Mieghem, C. E. Schotborgh, R. Hermanides, F. van der Kley, S. Kats, F. Porta, M. G. Stoel, G. Amoroso, M. van Wely, L. Timmers, M. Voskuil, H. W. van der Werf, Cardiothoracic Surgery Registry Committee of the Netherlands Heart Registry, S. Bramer, W. J. P. van Boven, A. B. A. Vonk, B. M. J. A. Koene, J. A. Bekkers, G. J. F. Hoohenkerk, A. L. P. Markou, A. de Weger, P. Segers, F. Porta, R. G. H. Speekenbrink, W. Stooker, W. W. L. Li, E. J. Daeter, N. P. van der Kaaij, Y. Douglas

**Affiliations:** 1 Department of Cardiothoracic Surgery and Cardiology, Catharina Hospital, Eindhoven, The Netherlands; 2 Department of Cardiothoracic Surgery and Cardiology, St. Antonius Hospital, Nieuwegein, The Netherlands; 3 Department of Cardiothoracic Surgery, Leiden University Medical Centre, Leiden, The Netherlands; 4 Netherlands Heart Registration, Utrecht, The Netherlands

**Keywords:** SAVR, TAVI, Indication document, NHR

## Abstract

**Introduction:**

Based on European guidelines, transcatheter aortic valve implementation (TAVI) could be the therapy of choice in patients with severe aortic stenosis aged ≥ 75 years. In the Netherlands, there has been a debate between healthcare providers and the National Health Care Institute regarding reimbursement for TAVI, which resulted in an indication document that defines TAVI patients who are eligible for reimbursement. This document has been effective since 1 January 2021.

**Methods:**

We extracted data from the Netherlands Heart Registry for patients who underwent biological surgical aortic valve replacement (SAVR) or TAVI in the Netherlands from 2018 through 2021. We compared baseline characteristics and variables from the indication document for the subsequent years and age groups. We also analysed the annual SAVR/TAVI ratio.

**Results:**

The total number of patients treated with SAVR or TAVI was constant in 2018–2021. Baseline characteristics of patients treated with TAVI did not differ throughout the years. The SAVR/TAVI ratio shifted towards a higher percentage of TAVI from 2018 to 2019. From 2019 to 2020, the TAVI percentage was constant. Since the implementation of the indication document (in 2021), a change in the SAVR/TAVI ratio was not found either.

**Conclusion:**

Since the implementation of the national indication document for AVR in 2021, no major effect was seen for the SAVR versus TAVI landscape in the Netherlands.

**Supplementary Information:**

The online version of this article (10.1007/s12471-023-01811-1) contains supplementary material, which is available to authorized users.

## What’s new?

- Evaluation of the impact of the Dutch national indication document since its implementation on 1 January 2021 showed it did not impact the surgical aortic valve replacement (SAVR) and transcatheter aortic valve implementation (TAVI) landscape in the Netherlands.
- The SAVR/TAVI ratio had already shifted in 2019, in favour of TAVI.

## Introduction

Aortic stenosis is the most prevalent valvular heart lesion requiring surgical aortic valve replacement (SAVR) or transcatheter aortic valve implementation (TAVI) in Europe and the United States [1]. As a consequence of aging, its prevalence is rising [2]. In the pre-TAVI era, patients at high surgical risk had no access to alternative treatment when surgical risk of death was unacceptably high. TAVI has been developed as a less invasive therapeutic alternative for such patients, to improve their quality of life and prognosis. In the past years, significant improvements in TAVI treatment and subsequent improvements in outcome compared with SAVR have led to increasing adoption of TAVI [3–5].

Generally, the indication for SAVR or TAVI is assessed by the Heart Team (consisting of at least a cardiologist and cardiothoracic surgeon), which includes an all-around risk assessment and an evaluation of the feasibility of the procedure. Initially, only high-risk patients were selected for TAVI based on available data [6, 7]. However, several trials made it possible to select patients for TAVI who had intermediate risk (PARTNER and SURTAVI), and even low risk (PARTNER 3 and Evolut PRO trials).

In 2020, the Dutch National Health Care Institute (*Zorginstituut Nederland*) published a report stating that TAVI should no longer be reimbursed for low- and intermediate-risk patients [8]. The institute requested the Transcatheter Heart Valve Intervention (THI) working group (consisting of cardiologists and cardiothoracic surgeons who are all members of the Netherlands Society of Cardiology (*Nederlandse Vereniging Voor Cardiologie*) or Netherlands Society of Thoracic Surgery (*Nederlandse Vereniging voor Thoraxchirurgie*)) to draft a document (hereafter: indication document) to define the—to be reimbursed—high-risk population for TAVI. This document was based on the 2017 European Society of Caddiology (ECS)/European Association for Cardio-Thoracic Surgery (EACTS) Guidelines for the management of valvular heart disease [6, 9] and available data from clinical trials at that time. This indication document was implemented by Heart Teams of all heart centres in the Netherlands on 1 January 2021. As a result, TAVI will only be reimbursed dependent on the patient’s age and the presence of significant comorbidities, as defined in the document. However, in the 2021 ESC/EACTS Guidelines, TAVI is recommended in patients ≥ 75 years of age, irrespective of the absence or presence of comorbidities [10].

The goal of this study was to evaluate the impact of the indication document on the SAVR and TAVI landscape in the Netherlands, since its implementation in 2021. We therefore evaluated the numbers of procedures and the ratio of SAVR to TAVI, in relation to age and throughout time.

## Methods

### Data collection

Patient and procedural characteristics and outcome variables were collected from the Netherlands Heart Registry (*Nederlandse Hart Registratie*, NHR). The NHR collects data from all 16 Dutch heart centres [11]. Data analysis for the current objective was approved by the NHR, registry committees for cardiology and cardiothoracic surgery and the THI working groups for cardiology and cardiothoracic surgery. The variables in the indication document [9] are registered since its implementation in January 2021 and were provided by the NHR. Data for some variables in the indication document could be derived from the NHR data for 2018–2020 (see Table S1 in Electronic Supplementary Material).

### Population

This retrospective national study comprised patients who underwent SAVR or TAVI for severe symptomatic aortic stenosis in 2018–2021. Patients with other significant valvular heart disease, an indication for concomitant cardiac surgery or recent (past 3 months) percutaneous coronary intervention or coronary artery bypass grafting were excluded from this study.

### Statistical analysis

Continuous variables are presented as median with interquartile range (IQR). Categorical variables are presented as count with percentage. Continuous variables were analysed with the Kruskal-Wallis test or Mann-Whitney U test, whichever was appropriate. Categorical variables were analysed using the chi-square test, Fisher’s exact test or Mann-Whitney U test, whichever was appropriate. Patient characteristics and procedures were compared for the subsequent years, as well as for different age cohorts (all ages, < 75 years, 75–80 years and ≥ 80 years). SPSS Statistics software version 26.0 (IBM Corp., Armonk, NY, USA) was used for the statistical analyses.

## Results

### Baseline characteristics

A total of 18,008 patients who met the inclusion criteria were included in this study. Of them, 9029 were treated with SAVR and 8979 with TAVI. Median age was 71.0 years (IQR: 64.0–75.0) in the SAVR cohort and 80.0 years (IQR: 76.0–84.0) in the TAVI cohort. Patients who underwent TAVI were older, were more frequently female and had a higher EUROSCORE I or II compared with the SAVR cohort. In the TAVI cohort, baseline characteristics did not differ significantly from 2018 through 2021. Table 1 shows all baseline characteristics with corresponding *p* values for both the SAVR and TAVI cohorts over time.

**Table 1:** Baseline characteristics

Characteristic	All procedures, all years (*N* = 18,008)	SAVR, all years (*n* = 9029)	TAVI, all years (*n* = 8979)	*P* value^a^	TAVI, 2021 (*n* = 2365)	TAVI, 2018/2019/2020 (*n* = 6614)	*P* value^b^
Age, years	75.0 (69.0–81.0)	71.0 (64.0–75.0)	80.0 (76.0–84.0)	< 0.001	81.0 (75.0–84.0)	80.0 (76.0–84.0)	0.339
Male	10,758 (59.7)	6174 (68.4)	4584 (50.8)	< 0.001	1213 (51.3)	3371 (51.0)	0.788
Body mass index, kg/m^2^	26.9 (24.3–30.1)	27.6 (24.7–30.4)	26.5 (23.9–29.8)	< 0.001	26.5 (23.9–29.5)	26.5 (23.9–30.0)	0.564
Diabetes mellitus	4212 (23.5)	1863 (20.6)	2349 (26.0)	< 0.001	579 (24.5)	1770 (26.8)	0.024
Diabetes mellitus on insulin	1367 (7.6)	584 (6.5)	783 (8.7)		198 (8.4)	585 (8.9)	
Chronic pulmonary disease	2637 (14.7)	1011 (11.2)	1626 (18.0)	< 0.001	428 (18.1)	1198 (18.1)	0.929
Prior cerebrovascular accident	1463 (8.1)	561 (6.2)	902 (10.0)	< 0.001	256 (10.8)	646 (9.8)	0.156
Atrial fibrillation	1231 (6.8)	1231 (13.6)	NA	NA	NA	NA	NA
Neurological dysfunction	474 (2.6)	154 (1.7)	320 (3.5)	< 0.001	88 (3.7)	232 (3.5)	0.800
Creatinine, µmol/l	87.0 (73.0–105.0)	84.0 (72.0–99.0)	90.0 (74.0–112.5)	< 0.001	89.0 (73.0–111.0)	91.0 (74.0–113.0)	0.139
Left ventricular ejection fraction, %	55.0 (50.0–55.0)	55.0 (55.0–55.0)	55.0 (43.0–55.0)	< 0.001	55.0 (44.0–55.0)	55.0 (44.0–55.0)	0.013
Mean right ventricular systolic pressure, mm Hg	25.0 (25.0–25.0)	25.0 (25.0–25.0)	25.0 (25.0–32.0)	< 0.001	25.0 (25.0–37.0)	30.0 (25.0–30.0)	0.198
*NYHA class*				< 0.001			< 0.001
– I	2786 (15.5)	1904 (21.1)	882 (9.8)		178 (7.5)	704 (10.6)	
– II	7100 (39.4)	4147 (45.9)	2953 (32.7)		934 (39.5)	2019 (30.5)	
– III	6644 (36.9)	2383 (26.4)	4261 (47.2)		1104 (46.7)	3157 (47.7)	
– IV	852 (4.7)	301 (3.3)	551 (6.1)		145 (6.1)	406 (6.1)	
Euroscore I	7.3 (4.3–12.4)	4.7 (3.0–7.1)	11.1 (7.8–17.0)	< 0.001	11.3 (7.9–16.8)	11.0 (7.7–17.1)	0.297
Euroscore II	2.3 (1.4–4.2)	1.7 (1.1–3.0)	3.3 (2.0–5.5)	< 0.001	3.1 (2.0–5.4)	3.2 (2.0–5.6)	0.659

Data are median (interquartile range) or *n* (%)

*NYHA* New York Heart Association, *NA* not applicable

^a^*P* value for comparison of surgical aortic valve replacement (S*AVR*) versus transcatheter aortic valve implementation (*TAVI*) for all years

^b^*P* value for comparison of TAVI in 2021 versus TAVI for all other years (2018, 2019 and 2020)

### Results for 2021 and earlier

**Fig. 1:**
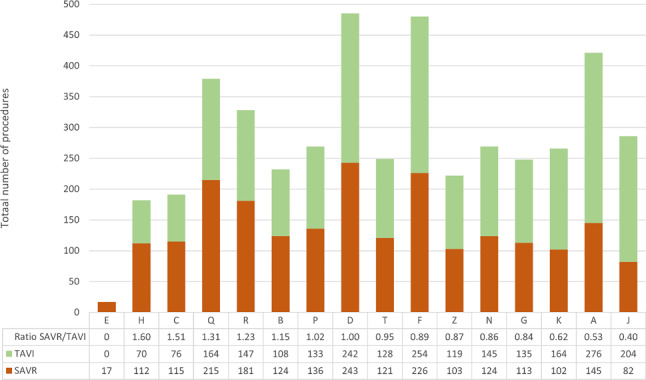
Ratio of surgical aortic valve replacement (*SAVR*) versus transcatheter aortic valve implementation (*TAVI*) per heart centre in 2021, with total number of procedures for SAVR and TAVI

The SAVR/TAVI ratio shifted towards a higher percentage of TAVI from 2018 to 2019 (45.3% (2014/4443) in 2018 vs 50.7% (2388/4714) in 2019; *p* < 0.001). For 2020 and 2021, the percentages of TAVI were comparable (51.1% (2212/4327) vs 52.3% (2365/4524)). The *p* values for these ratios were 0.66 (2019 vs 2020) and 0.28 (2020 vs 2021). In Fig. 1, the SAVR/TAVI ratios are displayed separately for all 16 heart centres in the Netherlands in 2021. This year, centre J had the lowest SAVR/TAVI ratio (71% of procedures was TAVI), whereas the highest ratio was observed in centre H (38% of procedures was TAVI). Additional SAVR/TAVI ratios are displayed in Table S2 in the Electronic Supplementary Material, showing centre data for all years.

**Fig. 2:**
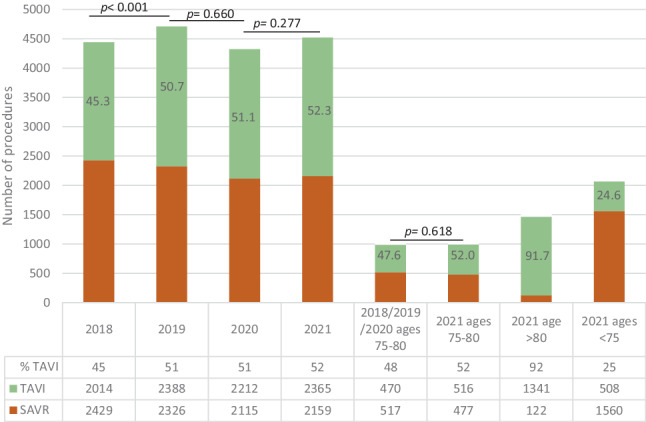
Number of transcatheter aortic valve implementation (*TAVI*) and surgical aortic valve replacement (*SAVR*) procedures in 2018–2021

Specific analysis per age group for 2021 (i.e., the year the indication document was introduced and the new European guidelines were published) showed that 52.0% of the patients in the age cohort 75–80 years were treated with TAVI. In the cohort < 75 years, 24.6% were treated with TAVI, whereas 91.7% received this treatment in the cohort ≥ 80 years (Fig. 2).

### Completeness of indication document variables

The indication document was mandatory for patients who underwent TAVI from 1 January 2021 and onwards and for patients who underwent SAVR from 1 January 2022 and onwards. The reported completeness of the indication document based on the NHR data for patients who underwent TAVI was 85.1% for 2021 and > 90% for 2022 [12].

### Variables in indication document

**Fig. 3:**
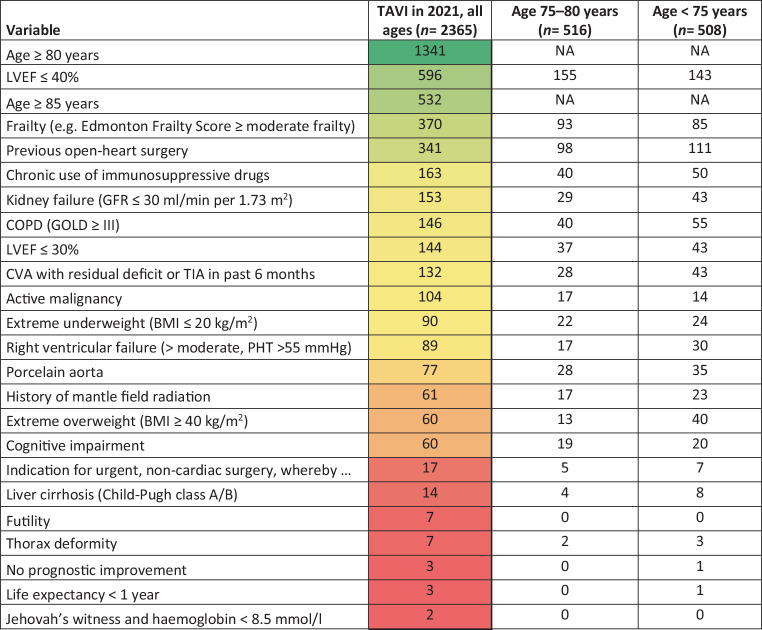
Most chosen variables of indication document resulting in TAVI. (*TAVI* transcatheter aortic valve implementation, *NA* not applicable, *LVEF* left ventricular ejection fraction, *GFR* glomerular filtration rate, *COPD* chronic obstructive pulmonary disease, *CVA* cerebrovascular accident, *TIA* transient ischaemic attack, *BMI* body mass index, *PHT* pulmonary hypertension)

The most commonly used variables that resulted in TAVI for the cohort all ages were ‘age ≥ 80 years’ (1341/2365; 56.7%), reduced left ventricular ejection fraction (LVEF) ≤ 40% (596/2365; 25.2%), ‘age ≥ 85 years’ (532/2365; 22.5%), more than moderate frailty (370/2365; 15.7%), previous cardiac surgery (open-heart surgery) (341/2365; 14.4%) and chronic use of immunosuppressive drugs (163/2365; 6.9%) (Fig. 3). For the cohorts 75–80 years and < 75 years, the most frequently used criteria were reduced LVEF ≤ 40% (155/516 (30.0%) and 143/508 (28.2%), respectively), previous cardiac surgery (98/516 (19.0%) and 111/508 (21.9%), respectively) and more than moderate frailty (93/516 (18.0%) and 85/508 (16.7%), respectively) (Fig. 3, and see Tables S3 and S4 in Electronic Supplementary Material).

From 2018 through 2021, patients in the age cohorts ≥ 80 years and ≥ 85 years were treated with TAVI more frequently in comparison with the other cohorts: 5004/5607 (89.2%) and 1952/2002 (97.5%), respectively. Of the patients with LVEF ≤ 40%, 58.9% (2115/3591) were treated with TAVI.

## Discussion

Since 2021, the Heart Teams of all Dutch 16 heart centres performing AVR procedures (SAVR and TAVI) have to base their decision for SAVR or TAVI in a specific patient upon the national indication document [8, 9]. This indication document defines which TAVI patients are eligible for reimbursement. The most prominent difference between the criteria in this indication document and the 2021 ESC/EACTS Guidelines for the management of valvular heart disease is that the latter recommends TAVI in patients aged ≥ 75 years [10], whereas the indication document only allows TAVI in patients < 80 years in case of significant comorbidity.

This analysis focused on the potential changes in the SAVR and TAVI landscape in the Netherlands after the implementation of the indication document. The total number of SAVR plus TAVI procedures and the SAVR/TAVI ratio did not differ in 2021 compared with 2019 and 2020. In addition, baseline characteristics for the TAVI cohort in 2021 did not differ from those in 2019 and 2020. One might therefore argue that the indication document did not change the landscape importantly. However, there are 2 important factors that should be taken into consideration.

First, the indication document might have dampened the potential increase in TAVI in 2021. In other words, without the indication document, what would have happened with the SAVR/TAVI ratio in 2021, after the publication of the new European guidelines in mid-2021? The new guidelines are based on trial data that were already available in 2019. We know that clinical experts generally adapt their practice rapidly, based on published results. In 2019 and 2020, the SAVR/TAVI ratio in the Netherlands was similar to that in 2021, which supports the notion that a potential effect in 2021 was not dampened by the indication document. If, in the most extreme case, all SAVR-treated patients in the age cohort 75–80 years in 2021 would instead have been treated with TAVI, this would have led to an annual increase of 477 TAVI procedures and the percentage of TAVI would have risen from 52 to 63% in 2021.

Second, the COVID-19 pandemic has affected hospital care, starting in March 2020. Did the pandemic create a potential bias with respect to this analysis? Rooijakkers and colleagues from the Radboud University Medical Centre in Nijmegen, the Netherlands showed the COVID-19 pandemic did not have an impact on TAVI patient characteristics and outcome [13]. In our analysis, we did not find arguments for a COVID-19 effect as the SAVR/TAVI ratios of 2018 (pre-COVID year) and 2020 (COVID year) did not differ, making a potential COVID effect less likely.

We analysed separate age cohorts in 2021 because of the discrepancy in age-based recommendations for TAVI between the European guidelines and the indication document (75 versus 80 years, respectively). Based on the criteria of the indication document, for the all age cohort, TAVI was more frequent in patients of older age (≥ 80 and ≥ 85 years), frail patients, patients with a reduced LVEF and those with previous cardiac surgery. These patients had a higher surgical risk profile with a higher risk of postoperative complications, making TAVI the preferred treatment. Rates of the variables ‘age ≥ 80 years’, ‘age ≥ 85 years’ and ‘reduced LVEF’ did not differ from 2018 through 2021. For the age group 75–80 years, similar findings were seen. This illustrates that consistent decisions have been made throughout the years and the indication document did not alter this.

### Study limitation

The indication document was implemented by Heart Teams in 2021. To see its full effect on Heart Team decisions, adding another full year could provide more insight into the annual number of procedures of TAVI versus SAVR.

## Conclusion

After implementation of the national indication document for AVR in the Netherlands in 2021, no major changes were seen in the SAVR versus TAVI landscape. Ongoing analysis of the impact of the indication document is warranted in the upcoming years.

### Supplementary Information

**Table S1** Variables in indication document

**Table S2** Number of procedures per centre plus SAVR/TAVI ratio

**Table S3** Variables in indication document for age group 75–80 years

**Table S4** Variables in indication document for age group < 75 years
