# Supplementary figures and images for: National indication document and aortic valve replacement landscape in the Netherlands
Source: Neth Heart J. 2023 Oct 16;31(12):473–8. doi: 10.1007/s12471-023-01811-1 (PMC10667164; doi:10.1007/s12471-023-01811-1)

**Table S1** Variables in indication document


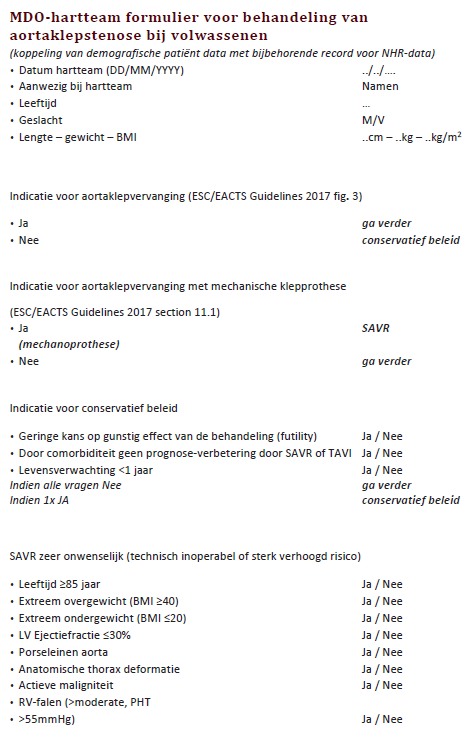


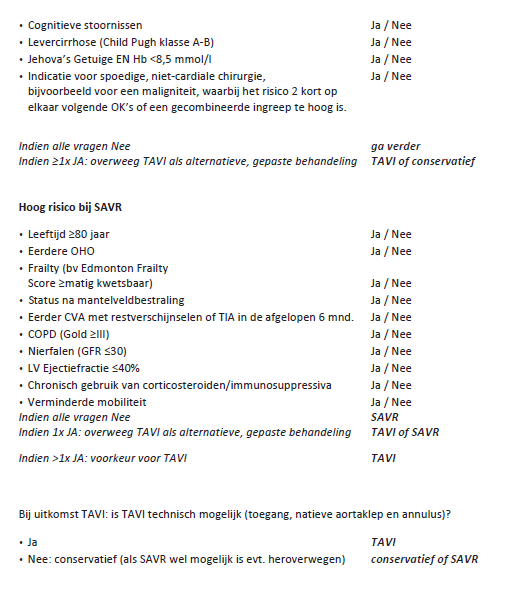

Supplement: Supplementary file 1 — Table S1 Variables in indication document [file 12471_2023_1811_MOESM1_ESM.docx]
