# Supplementary material for: National indication document and aortic valve replacement landscape in the Netherlands
Source: Neth Heart J. 2023 Oct 16;31(12):473–8. doi: 10.1007/s12471-023-01811-1 (PMC10667164; doi:10.1007/s12471-023-01811-1)
Supplement: Supplementary file 2 — Table S2 Number of procedures per centre plus SAVR/TAVI ratio [file 12471_2023_1811_MOESM2_ESM.docx]

**Table S2** Number of procedures per centre plus SAVR/TAVI ratio^a^

|  | SAVR | TAVI | Ratio | SAVR | TAVI | Ratio | SAVR | TAVI | Ratio | SAVR | TAVI | Ratio | SAVR | TAVI | Ratio | SAVR | TAVI | Ratio | SAVR | TAVI | Ratio |
| --- | --- | --- | --- | --- | --- | --- | --- | --- | --- | --- | --- | --- | --- | --- | --- | --- | --- | --- | --- | --- | --- |
| Centre | Total |  |  | 2018 |  |  | 2019 |  |  | 2020 |  |  | 2021 |  |  | 2021 Ages 75-80 | | | 2021 Ages < 75 | |  |
| A | 442 | 1020 | 0.43 | 116 | 262 | 0.44 | 88 | 247 | 0.36 | 93 | 235 | 0.40 | 145 | 276 | 0.53 | 35 | 53 | 0.66 | 109 | 59 | 1.85 |
| B | 438 | 357 | 1.23 | 96 | 70 | 1.37 | 118 | 90 | 1.31 | 100 | 89 | 1.12 | 124 | 108 | 1.15 | 33 | 26 | 1.27 | 86 | 22 | 3.91 |
| C | 414 | 343 | 1.21 | 108 | 81 | 1.33 | 92 | 103 | 0.89 | 99 | 83 | 1.19 | 115 | 76 | 1.51 | 30 | 14 | 2.14 | 76 | 17 | 4.47 |
| D | 1163 | 818 | 1.42 | 345 | 137 | 2.52 | 312 | 234 | 1.33 | 263 | 205 | 1.28 | 243 | 242 | 1.00 | 60 | 54 | 1.11 | 179 | 50 | 3.58 |
| E | 303 | 114 | 2.66 | 139 | 35 | 3.97 | 92 | 62 | 1.48 | 55 | 17 | 3.24 | 17 | 0 | xxx | 4 | 0 | xxx | 13 | 0 | xxx |
| F | 935 | 849 | 1.10 | 256 | 167 | 1.53 | 242 | 205 | 1.18 | 211 | 223 | 0.95 | 226 | 254 | 0.89 | 47 | 50 | 0.94 | 161 | 56 | 2.88 |
| G | 405 | 506 | 0.80 | 106 | 132 | 0.80 | 89 | 127 | 0.70 | 97 | 112 | 0.87 | 113 | 135 | 0.84 | 14 | 38 | 0.37 | 93 | 39 | 2.38 |
| H | 496 | 233 | 2.13 | 135 | 47 | 2.87 | 134 | 54 | 2.48 | 115 | 62 | 1.85 | 112 | 70 | 1.60 | 26 | 14 | 1.86 | 69 | 8 | 8.63 |
| J | 454 | 817 | 0.56 | 141 | 182 | 0.77 | 129 | 226 | 0.57 | 102 | 205 | 0.50 | 82 | 204 | 0.40 | 12 | 52 | 0.23 | 70 | 56 | 1.25 |
| K | 383 | 574 | 0.67 | 99 | 135 | 0.73 | 83 | 166 | 0.50 | 99 | 109 | 0.91 | 102 | 164 | 0.62 | 22 | 38 | 0.58 | 74 | 35 | 2.11 |
| N | 328 | 530 | 0.62 | 1 | 124 | 0.01 | 96 | 135 | 0.71 | 107 | 126 | 0.85 | 124 | 145 | 0.86 | 23 | 28 | 0.82 | 100 | 40 | 2.50 |
| P | 596 | 554 | 1.08 | 176 | 131 | 1.34 | 166 | 146 | 1.14 | 118 | 144 | 0.82 | 136 | 133 | 1.02 | 40 | 27 | 1.48 | 88 | 17 | 5.18 |
| Q | 933 | 703 | 1.33 | 251 | 176 | 1.43 | 235 | 184 | 1.28 | 232 | 179 | 1.30 | 215 | 164 | 1.31 | 48 | 34 | 1.41 | 141 | 21 | 6.71 |
| R | 774 | 554 | 1.40 | 193 | 121 | 1.60 | 214 | 139 | 1.54 | 186 | 147 | 1.27 | 181 | 147 | 1.23 | 44 | 30 | 1.47 | 124 | 26 | 4.77 |
| T | 474 | 530 | 0.89 | 119 | 118 | 1.01 | 112 | 139 | 0.81 | 122 | 145 | 0.84 | 121 | 128 | 0.95 | 17 | 33 | 0.52 | 101 | 30 | 3.37 |
| Z | 491 | 477 | 1.03 | 148 | 96 | 1.54 | 124 | 131 | 0.95 | 116 | 131 | 0.89 | 103 | 119 | 0.87 | 22 | 25 | 0.88 | 76 | 32 | 2.38 |

^a^ Red colour indicates SAVR/TAVI ratio > 1; green colour indicates SAVR/TAVI ratio < 1
