# Supplementary material for: National indication document and aortic valve replacement landscape in the Netherlands
Source: Neth Heart J. 2023 Oct 16;31(12):473–8. doi: 10.1007/s12471-023-01811-1 (PMC10667164; doi:10.1007/s12471-023-01811-1)
Supplement: Supplementary file 3 — Table S3 Variables in indication document for age group 75–80 years [file 12471_2023_1811_MOESM3_ESM.docx]

**Table S3** Variables in indication document for age group 75–80 years

|  | TAVI year 2021 ages 75-80 |
| --- | --- |
| Left ventricular ejection fraction ≤40% | 155 |
| Previous OHO | 98 |
| Frailty (ex. Edmonton Frailty Score ≥ moderate frailty) | 93 |
| COPD (GOLD ≥III) | 40 |
| Chronic use immune suppressive drugs | 40 |
| Left ventricular ejection fraction ≤30% | 37 |
| Kidney failure (GFR ≤30) | 29 |
| Porcelain aorta | 28 |
| CVA with residual deficit or TIA in past 6 months | 28 |
| Active malignancy | 27 |
| Right ventricular failure (>moderate, PHT >55mmHg) | 22 |
| Cognitive impairment | 19 |
| Extreme underweight (BMI ≤20) | 17 |
| History of mantle field radiation | 17 |
| Extreme overweight (BMI ≥40) | 13 |
| Indication for urgent, non-cardiac surgery, whereby … | 5 |
| Liver cirrhosis (Child Pugh class A-B) | 4 |
| Thorax deformity | 2 |

* BMI: body mass index; CVA: cerebrovascular accident; GFR: glomerular filtration rate; OHO: open heart operation; PHT: pulmonary hypertension; TIA: transient ischemic attack
